# The Role of Anti-EGFR Monoclonal Antibody in mCRC Maintenance Therapy

**DOI:** 10.3389/fmolb.2022.870395

**Published:** 2022-03-30

**Authors:** Meiqin Yuan, Zeng Wang, Wangxia Lv, Hongming Pan

**Affiliations:** ^1^ The Cancer Hospital of the University of Chinese Academy of Sciences (Zhejiang Cancer Hospital), Institute of Basic Medicine and Cancer (IBMC), Chinese Academy of Sciences, Hangzhou, China; ^2^ Zhejiang University School of Medicine, Hangzhou, China; ^3^ Zhejiang University School of Medicine, Sir Run Run Shaw Hospital, Hangzhou, China

**Keywords:** mCRC, maintenance therapy, EGFR, monoclonal antibody, panitumumab, cetuximab

## Abstract

**Background:** Epidermal growth factor receptor (EGFR) monoclonal antibodies (mAbs) combined with chemotherapy in patients with RAS (rat sarcoma viral oncogene homolog) wild-type metastatic colorectal cancer (mCRC) can alleviate and stabilize the disease, effectively prolong the progression-free survival (PFS) and overall survival (OS), and improve the overall response rate (ORR), which is the first-line treatment standard scheme for RAS wild-type mCRC currently. However, whether anti-EGFR mAb can be used for the maintenance treatment after the first-line treatment of mCRC remains controversial. We reviewed the recent studies on anti-EGFR mAb. The contents include five parts, introduction, anti-EGFR mAb in mCRC and its status in first-line therapy, establishment of the maintenance treatment pattern after the standard first-line treatment for mCRC, research progress of anti-EGFR mAb in mCRC maintenance therapy, and conclusion. More studies support the maintenance treatment of anti-EGFR mAb, but some researchers raise the problems about high cost and drug resistance. Despite lack of the maintenance evidence of anti-EGFR mAb, especially lack of large-scale phase III prospective clinical trials, with the emergence of new evidence and more accurate screening of treatment-dominant groups, maintenance therapy with anti-EGFR mAb monotherapy or anti-EGFR mAb combined with fluorouracil-based schemes after first-line chemotherapy combined with anti-EGFR mAb therapy might strive for more treatment opportunities, optimize treatment strategies and prolong treatment continuity, and finally, lead to more survival benefit for suitable patients.

## 1 Introduction

**FIGURE 1:**
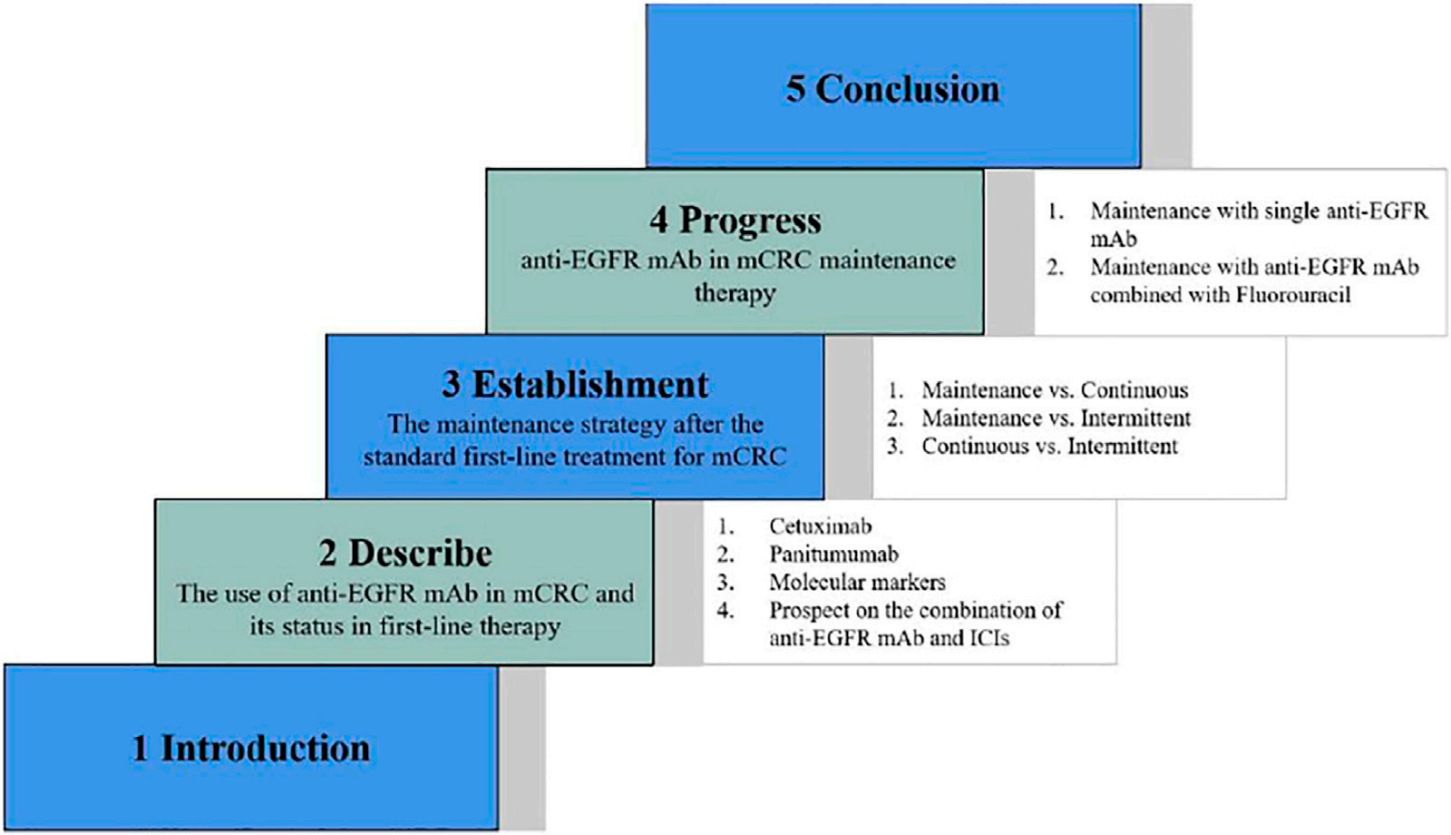
The scheme for the full text. The structure of this article is shown which consists of five parts, including introduction, description of the use of anti-EGFR mAb in mCRC as well as its status in first-line therapy, establishment of the maintenance strategy after the standard first-line treatment for mCRC, progress of anti-EGFR mAb in mCRC maintenance therapy, and conclusion.

Colorectal cancer is a serious threat to human life, and the epidemiological data in 2020 showed that its incidence rate was increasing year by year, accounting for the third place in the global cancer data and the second place in the Chinese cancer data (Siegel et al., 2020). Colorectal cancer has a high degree of malignancy and poor prognosis, and the data show that its mortality rate ranks third in global cancer and fifth in the Chinese cancer (Siegel et al., 2020). Metastatic colorectal cancer (mCRC) has a high mortality rate and lacks effective systemic treatments. Epidermal growth factor receptor (EGFR) monoclonal antibodies (mAbs) such as cetuximab and panitumumab combined with chemotherapy in patients with RAS wild-type mCRC can alleviate and stabilize the disease, effectively prolong the progression-free survival (PFS) and overall survival (OS), and improve the overall response rate (ORR), which is the first-line treatment standard scheme for RAS wild-type mCRC currently (Khattak et al., 2015; Stintzing et al., 2016). Maintenance therapy, which refers to continuous treatment with drugs of low intensity and toxicity after a period of first-line treatment that achieves optimal efficacy and is in a stable state of disease, is a common treatment modality used in the current treatment of mCRC. It can achieve the aims of prolonging the PFS, reducing adverse effects, delaying the time of recurrence of tumor-related symptoms, and improving the patients’ quality of life. However, whether cetuximab can be used for maintenance treatment after the first-line treatment of mCRC remains controversial. This article reviews the research progress of anti-EGFR mAb maintenance therapy in recent years. Figure 1 shows the scheme for the full text.

## 2 Anti-EGFR mAb in Metastatic Colorectal Cancer and Its Status in First-Line Therapy

**FIGURE 2:**
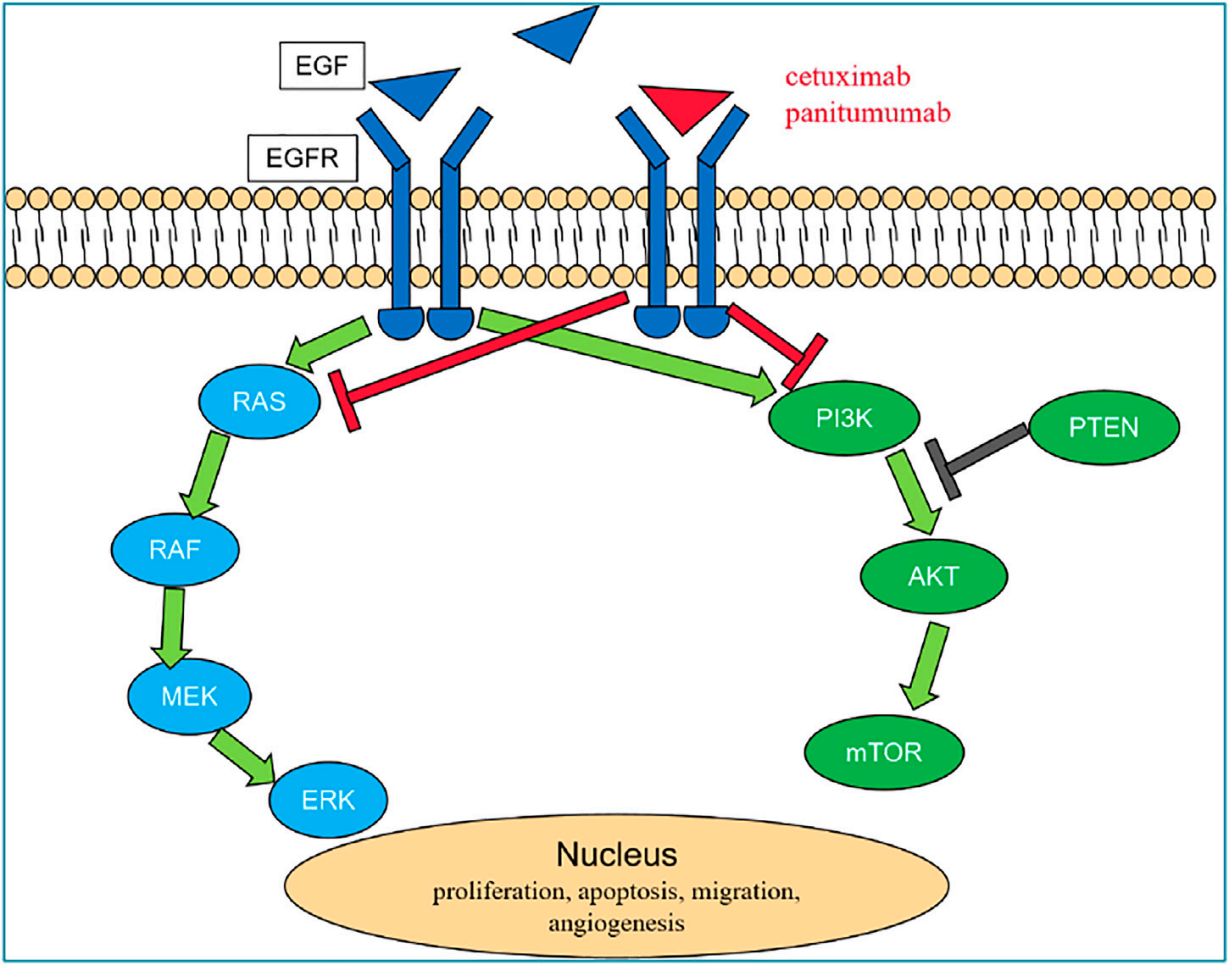
EGFR pathway. In malignant cells, EGF combine to EGFR and activate the EGFR pathway which initiates a downstream signaling cascade through the RAS/RAF/MAPK and the PI3K/AKT/mTOR axes and then promotes cell proliferation. Cetuximab and panitumumab are anti-EGFR mAbs, which by competitive binding to the extracellular domain of EGFR lead to the internalization, degradation of the receptor, and inhibition of the EGFR signal transduction pathway and also through the antibody-dependent cytotoxic effect inhibit the proliferation of tumor cells and induce apoptosis, so as to achieve the effect of tumor control. Abbreviations: AKT, AKT8 virus oncogene cellular homolog; EGFR, epidermal growth factor receptor; ERK, extracellular signal-regulated kinase; MEK, mitogen-activated protein kinase; mTOR, mammalian target of rapamycin; PI3K, phosphatidyilinositol 3-kinase; PTEN, phosphatase and tensin homolog; RAF, v-Raf murine sarcoma viral oncogene homolog; and RAS, rat sarcoma viral oncogene homolog.

EGFR, belonging to the tyrosine kinase-type receptor, is a member of the epidermal growth factor (ErbB) receptor family, which includes EGFR (HER1/ErbB-1, human epidermal growth factor-associated receptor), HER2 (ErbB-2), HER3 (ErbB-3), and HER4 (ErbB-4) (Yarden, 2001). EGFR is constitutively expressed in normal epithelial tissues. In malignant cells, the activation of the EGFR pathway initiates a downstream signaling cascade which promotes cell proliferation through the RAS (rat sarcoma viral oncogene homolog)/RAF (v-Raf murine sarcoma viral oncogene homolog)/MAPK (mitogen-activated protein kinase) and the PI3K (phosphatidylinositol 3-kinase)/AKT (AKT8 virus oncogene cellular homolog)/mTOR (mammalian target of rapamycin) axes (Hynes and Lane, 2005; Citri and Yarden, 2006). Studies have shown that EGFR is highly expressed or abnormally expressed in many solid tumors of epithelial origin, such as head and neck cancer and colorectal cancer, which is related to cancer cell proliferation, tumor angiogenesis, tumor invasion, and metastasis (Yamaoka et al., 2017). Chan et al. (2017) showed that anti-EGFR mAb combined with conventional chemotherapy can prolong the PFS and OS of mCRC patients and improve the tumor response rate (RR). For patients with RAS wild-type mCRC, the currently approved anti-EGFR mAbs are panitumumab and cetuximab; however, panitumumab has not yet been marketed in China. Cetuximab is a human–mouse chimeric IgG1 anti-EGFR mAb, and panitumumab is the first fully humanized anti-EGFR mAb, both targeting EGFR, by binding to the extracellular domain of the receptor, leading to the internalization, degradation, and inhibition of the EGFR signal transduction pathway, also through the antibody-dependent cytotoxic effect to inhibit the proliferation of tumor cells and induce apoptosis, so as to achieve the effect of tumor control (Figure 2). Clinical research data also support the application of anti-EGFR mAb in patients with advanced colorectal cancer.

### 2.1 Cetuximab

Cetuximab is a human–mouse chimeric IgG1 monoclonal antibody targeting EGFR. The phase III clinical trial CRYSTAL reported by Van-Cutsem et al. (2007) was designed to observe the effect of first-line cetuximab combined with chemotherapy in mCRC patients. A total of 1,217 newly treated mCRC patients with EGFR expression were included. The results suggest that compared with the FOLFIRI (irinotecan 180 mg/m^2^ d1, leucovorin 400 mg/m^2^ d1, and fluorouracil 400 mg/m^2^ bolus d1 and 2,400 mg/m^2^ civ over 46 h) group alone, the median progression-free survival (mPFS) of the combined cetuximab group was 0.9 months longer (8.9 vs. 8.0 months), which was statistically different, but the researchers were not satisfied. Further analysis of the correlation between the KRAS (Kirsten rat sarcoma viral oncogene) mutation status of tumor tissue and clinical efficacy showed that wild-type patients receiving cetuximab combined with FOLFIRI treatment had significantly better efficacy than the FOLFIRI group: RR was 59 vs. 43%, and mPFS was 9.9 vs. 8.7 months, whereas there was no significant difference between the two groups in the mutant population. The OPUS study (Van Cutsem et al., 2011) is an open-label, randomized, multicenter phase II study, which included 337 newly treated mCRC patients with EGFR-expressing metastatic colorectal cancer who were randomized to receive FOLFIRI alone or combined with cetuximab. The results showed that the objective efficacy was improved, but there was no difference in survival indicators. Similarly, KRAS mutation was performed. After the correlation analysis with clinical efficacy, it was found that the ORR and mPFS were significantly improved in KRAS wild-type patients receiving cetuximab combined with FOLFIRI. The COIN study (Maughan et al., 2011) is a phase III clinical trial comparing the efficacy of cetuximab combined with the FOLFOX4 (oxaliplatin 85 mg/m^2^ d1, leucovorin 200 mg/m^2^ d1–2, and fluorouracil 400 mg/m^2^ bolus and then 600 mg/m^2^ civ 22 h for d1–2) or XELOX (oxaliplatin 130 mg/m^2^ d1 and capecitabine 850–1,250 mg/m^2^ bid d1–14) regimen, as well as the FOLFOX4 or XELOX regimen alone, in the first-line treatment of patients with KRAS wild-type mCRC. The results showed that mCRC patients could benefit from the cetuximab combined with FOLFOX regimen. However, in combination with the XELOX regimen, the benefit was not obvious, and the overall survival (OS) of patients tends to be shortened compared with chemotherapy alone. The TAILOR study (Qin et al., 2018) compared the efficacy of the first-line use of FOLFOX4 + cetuximab and FOLFOX4 alone in patients with RAS wild-type mCRC. The results showed that the patients in the combination group were significantly better than those in the single-agent group in terms of PFS, and the first-line FOLFOX4 + cetuximab significantly improved the PFS, OS, and ORR of the Chinese RAS wild-type mCRC patients. The abovementioned studies have established the status of cetuximab combined with chemotherapy as the standard first-line treatment for patients with RAS wild-type advanced colorectal cancer.

### 2.2 Panitumumab

Panitumumab is a human anti-EGFR mAb antibody, which was approved for the treatment of RAS wild-type mCRC patients. The PRIME study (Douillard et al., 2010) is a phase III randomized clinical trial of panitumumab combined with FOLFOX4 versus FOLFOX4 alone in the first-line treatment of the untreated mCRC patients. A total of 1,183 patients were included, and 93% had KRAS results. In KRAS wild-type patients, panitumumab combined with FOLFOX4 significantly improved mPFS compared with FOLFOX4 alone, at 9.6 and 8.0 months, respectively, reaching a statistically significant difference. The PEAK study (Schwartzberg et al., 2014) compared the efficacy of panitumumab combined with chemotherapy versus bevacizumab combined with chemotherapy in patients with KRAS exon 2 wild-type mCRC. The results showed that there was no significant difference in the primary endpoint of the two groups for PFS. However, the combination with panitumumab had a 10-month advantage in OS compared with the combination with bevacizumab. These studies also confirmed the efficacy of panitumumab combined with chemotherapy as a first-line regimen in KRAS wild-type patients.

### 2.3 Molecular Markers Associated With the Anti-EGFR mAb

The participation of anti-EGFR mAbs such as cetuximab and panitumumab has improved the overall survival of the mCRC. However, the response rate of the anti-EGFR mAb-containing regimen is generally lower than 30% in unselected patient populations. Clinical data had confirmed the predictive value of RAS mutations to cetuximab and panitumumab resistance, resulting in the approval of these monoclonal antibodies only for the treatment of patients with RAS wild-type colorectal cancer. However, studies on the identification of predictive biomarkers are still ongoing.

#### 2.3.1 KRAS/Neuroblastoma Rat Sarcoma Viral Oncogene (NRAS)

RAS mutations can be detected in about 50% of the colorectal cancer patients, of which KRAS mutation rate is 40% and NRAS mutation rate is 3–5%, and HRAS mutations are very rare. Studies showed that KRAS mutations can result in sustained activation of EGF/RAS/RAS/RAF/ERK signaling the independence of EGFR and patients exhibiting ineffectiveness to anti-EGFR mAb or even deleterious. The value of mutations of KRAS exon 2 in predicting the resistance to panitumumab and panitumumab was confirmed by the clinical data (Rafael et al., 2008; Van Cutsem et al., 2009). Alterations in exons 2, 3, and 4 of either gene of the KRAS and NRAS both constitutively activate the RAS and are mutually exclusive. To date, several retrospective, non-prespecified analyses of randomized clinical trials have confirmed that the pan-RAS mutation acts as a negative predictor for anti-EGFR therapy (Douillard et al., 2013; Heinemann et al., 2014). However, patients with RAS wild-type mCRC could clearly benefit from the anti-EGFR treatment, with a significantly prolonged overall survival time. Especially in patients with left-half CRC, chemotherapy combined with anti-EGFR mAb had an mOS up to 55 months or more. However, the role of KRAS G13D mutations in primary resistance to anti-EGFR treatment remains controversial. It has been shown that patients carrying the KRAS G13D mutation may benefit from cetuximab treatment, and patients with KRAS G13D mutations had longer overall survival time (overall survival, OS) and progression-free survival time (progression free survival, PFS) compared to those with other KRAS mutations (the mOS was 7.6 and 5.7 months, respectively, HR = 0.50, *p* = 0.004; the mPFS was 4.0 and 1.9 months, respectively, HR = 0.51, *p* = 0.005). It suggested that the patients with KRAS G13D mutations may benefit from cetuximab therapy (De Roock et al., 2010a). However, a recent retrospective analysis showed no significant difference in OS (mOS was 8.2 and 14.6 months, respectively, HR = 0.50, *p* = 0.084) and PFS (mPFS was 4.96 and 3.1 months, HR = 0.88, *p* = 0.72, respectively) (Peeters et al., 2013). Therefore, the role of KRAS G13D mutations in primary resistance to anti-EGFR therapy remains controversial. Drugs targeting KRAS G13D mutations are currently under development.

#### 2.3.2 v-Raf Murine Sarcoma Viral Oncogene Homolog B1 (BRAF)

Not all RAS wild-type patients respond well to the anti-EGFR treatment. It is well known that BRAF is an important transduction factor in the EGFR signaling pathway RAS/RAF/MEK/MRK/MAPK and is involved in the regulation of various physiological processes such as cell growth, differentiation, and apoptosis. Approximately 5–9% of CRC patients carry BRAF V600E allele mutations, and multiple studies suggest that mutations in BRAF V600E can lead to the activation of persistent downstream signaling pathways leading to cell proliferation or survival and suggesting poor prognosis (Guedes et al., 2013; Li et al., 2013). A retrospective clinical data from 11 centers in Europe analyzed the effect of BRAF mutations on the efficacy of using cetuximab-combined chemotherapy in patients with chemo-refractory mCRC. The results showed that the patients with BRAF V600E mutation had a significantly reduced ORR compared with the wild-type patients (8.3 and 38.0%, OR = 0.15, *p* = 0.0012) (De Roock et al., 2010b). Therefore, clinicians should also fully consider the presence of BRAF mutations in the tumor before using anti-EGFR treatment.

#### 2.3.3 Other Molecular Markers

RAS and BRAF are currently the most studied and relatively well-defined predictive biomarkers of anti-EGFR therapy, and in addition to the RAS/RAF axis, EGFR also triggers the PIK3CA/PTEN signaling pathway. Molecular alterations of this pathway can lead to downstream signaling pathway activation through mechanisms unrelated to EGFR. Therefore, the role of PIK3CA/PTEN signaling activation in anti-EGFR therapeutic resistance is worth exploring. In addition, although the HER2 mutation or amplification gene occurs less frequently in colorectal cancer, the HER2 mutation or amplification may also predict the resistance to cetuximab (Kavuri et al., 2015). Additional biomarkers predicting the sensitivity of cetuximab are currently being explored, such as vascular endothelial growth factor 2 (VEGF2), epithelial–stromal conversion factor, fibroblast growth factor receptor, epidermodulin, and miR-31-3p (Sartore-Bianchi et al., 2016; Sunakawa et al., 2016; Mizukami et al., 2017; Pugh et al., 2017).

In current clinical practice, anti-EGFR mAb is only approved for patients with RAS wild-type colorectal cancer. However, after treatment with anti-EGFR mAb, the vast majority of RAS wild-type colorectal cancer patients will develop disease progression within 3–12 months of treatment (Emburgh et al., 2014), which is mainly caused by the abnormal activation of the signaling cascade downstream of EGFR, including alterations of member genes in the RAS/RAF, PIK3CA/PTEN, and JAK/STAT pathway and activation of selective growth factor receptor bypass such as IGF1R, HER2, and MET. This could explain more than 70% of the colorectal cancer cases with ineffective anti-EGFR treatment. Therefore, more clinical trials are needed to explore whether other biomarkers can be effectively applied in clinical practice. Thus, a deeper definition of anti-EGFR mAb resistance mechanisms will facilitate the development of new therapeutic strategies to overcome the primary and acquired resistance of anti-EGFR mAb therapy.

### 2.4 Prospect on the Combination of Anti-EGFR mAb and ICIs (Immune Checkpoint Inhibitors)

With the advent of the era of tumor immunotherapy, anti-EGFR therapy of colorectal cancer has also ushered in great opportunities, such as whether ICIs which include anti-PD-1(Programmed Cell Death 1)/PDL-1 (Programmed Cell Death Ligand 1)/CTLA-4 (Cytotoxic T-Lymphocyte-Associated Protein 4) antibody combined to anti-EGFR treatment could improve effectiveness and translate into patient survival benefits further. Preclinical studies have shown that anti-EGFR therapy induces tumor-specific immune responses and immunogenic apoptosis, while functional adaptive immunity mediates the effect. However, resistance to anti-EGFR therapy is also inevitable, and resistance is associated with an increased expression of CTLA-4 and PD-L1. Based on the complementary and synergistic activities of anti-EGFR mAb and ICIs, researchers believe that this is the basis for the combination of ICIs and EGFR mAb (Ferris et al., 2018). A phase II, single-arm, multicenter Simon two-phase trial of panitumumab plus ipilimumab plus nivolumab in RAS/BRAF wild-type, MSS (microsatellite stable) mCRC who received 1–2 prior lines of therapy and no prior anti-EGFR mAb and ICIs was carried out. In 49 evaluable subjects, the response rate at 12 weeks was 35% and reached the primary study endpoint. Also, the mPFS was 5.7 months (95% CI 5.5–7.9), better than the data for the previous anti-EGFR mAb monotherapy. The results suggest the activity of the combination of ICIs and anti-EGFR mAb in MSS mCRC (Lee et al., 2021). AVETUX is a single-arm phase II trial, which combined mFOLFOX6 and cetuximab with avelumab in RAS/BRAF wild-type and previously untreated mCRC patients, until secondary excision, disease progression, or toxicity. A total of 43 patients were enrolled. The primary endpoint 12-month progression-free survival rate was 40%, was inferior to the target endpoint (57%). The ORR was 79.5%; disease control rate was 92.3%; and early tumor shrinkage (ETS) rate (≥20% after 8 weeks) was 79.5% (Alexander et al., 2020). Researchers announced the results of the interim analysis of the AVETUXIRI study in the 2021 ASCO-GI meeting. This is a proof-of-concept, open-label, nonrandomized phase IIa study of avelumab in combination with anti-EGFR mAb and irinotecan in treatment-resistant MSS mCRC. The results showed that the ORR of the RAS wild-type (WT) group was 30%, which reached the preset threshold, and the phase II study could be continued. PR was not observed in the RAS mutation (MUT) group, but DCR was 60 and 61.5% in RAS WT and RAS MUT groups, respectively, PFS was 4.2 and 3.8 months, and OS was 12.7 and 14.0 months, respectively. The 6-month PFS rates were 40.0 and 38.5%, respectively. This study also indicates that there is a great space for exploration in the field of the combination of ICIs and EGFR mAb in mCRC (Van Den Eynde et al., 2021). CAVE, which was a single-arm, multicenter phase II trial, enrolled patients with RAS WT mCRC patients who had a complete or partial response to the first-line chemotherapy plus anti-EGFR drugs, developed acquired resistance, and failed the second-line therapy. The study met the primary end point, with an mOS of 11.6 months (95% CI, 8.4–14.8 months). mPFS was 3.6 months (95% CI, 3.2–4.1 months). Patients with RAS/BRAF WT ctDNA (circle tumor deoxyribonucleic acid) had an mOS of 17.3 months (95% CI, 12.5–22.0 months) compared with 10.4 months (95% CI, 7.2–13.6 months) in patients with mutated ctDNA (95% CI, 0.27–0.90; *p* = 0.02). The mPFS was 4.1 months (95% CI, 2.9–5.2 months) in RAS/BRAF WT patients compared with 3.0 months (95% CI, 2.6–3.5 months) in patients with mutated ctDNA (95% CI, 0.23–0.75; *p* = 0.004). This trial suggests that cetuximab plus avelumab is an active therapy in RAS WT mCRC. Also, plasma ctDNA analysis before the treatment may screen off patients who could benefit (Martinelli et al., 2021).

At present, there are still a number of clinical studies on anti-EGFR mAb combined with ICIs in the treatment of mCRC, which will provide more evidence to support their combination. Furthermore, genetic testing of the rebiopsied tumor tissue or peripheral blood may accurately guide the next step of treatment.

## 3 Establishment of the Maintenance Treatment Pattern After the Standard First-Line Treatment for Metastatic Colorectal Cancer

For advanced colorectal cancer patients who benefit from the standard first-line treatment, the next step is continuous treatment with the induction regimen, discontinuation of the induction regimen, or maintenance treatment with certain low-toxic drugs. In view of this question, domestic and foreign scholars have conducted a series of studies.

### 3.1 Maintenance Treatment vs. Continuous Treatment

Maintenance therapy refers to the continuous use of low-intensity and low-toxicity drugs after a period of first-line treatment, when the optimal efficacy is achieved and the disease is in a stable state. Also, continuous treatment means standard treatment until disease progression or intolerable toxicity. What is the outcome of maintenance treatment compared with continuous treatment? The OPTIMOX1 study (Tournigand et al., 2006) was an international multicenter randomized clinical trial, which enrolled advanced colorectal cancer patients with six-cycle FOLFOX (leucovorin, fluorouracil, and oxaliplatin) induction. Patients were randomized into two groups: the continuous group (group A) was continuously treated with the FOLFOX protocol, and the maintenance group (group B) received treatment with 5-fluorouracil and leucovorin (5-FU/LV); all patients were treated until disease progression or intolerable toxic side effects. A total of 620 patients were included, and the results showed that mPFS and median OS (mOS) were 9.0 and 19.3 months, respectively, in group A and 8.7 and 21.2 months, respectively, in group B. There was no significant difference in PFS or OS between the two groups, but the maintenance group was safer than the continuous group; especially in neurotoxicity, grade 3 neurotoxicity group A vs. B: 17.9 vs. 13.3%. The MACRO TTD study (Díaz-Rubio et al., 2012) was a multicenter, randomized, open-label phase III clinical study, with 480 advanced colorectal cancer patients randomized to the single-agent bevacizumab maintenance therapy or XELOX + BEV continuous therapy after a six-cycle first-line XELOX + BEV induction. The results showed that the mPFS was 10.4 months in the maintenance group and 9.7 months in the continuous group, and the mOS was 23.2 months in the maintenance group and 20.0 months in the continuous group, respectively. None of them showed statistically significant differences, but the security of the maintenance group is better. The STOP and GO study (Yalcin et al., 2013) was slightly different from the OPTIMOX1 and MACRO TTD study, which adopted a combination with capecitabine and bevacizumab for maintenance therapy. The mPFS was better in the maintenance treatment group, compared with the continuous treatment group, 11.0 vs. 8.3 months (*p* = 0.002), but there was still no significant difference in OS between the two groups. It can be seen from the abovementioned results that compared with continuous treatment, maintenance treatment with 5-FU monotherapy or bevacizumab monotherapy or combination with capecitabine and bevacizumab can improve economic benefits and patient quality of life without losing OS and, thus, has a certain clinical significance.

### 3.2 Maintenance Treatment vs. Intermittent Treatment

Different from maintenance treatment, intermittent treatment refers to complete discontinuation of all treatments after a period of standard treatment. After the OPTIMOX1 study, the researcher started the OPTIMOX2 study (Chibaudel et al., 2009). After induction treatment with the FOLFOX protocol, patients were divided into a fluorouracil maintenance treatment group and intermittent treatment group. The result indicated that mPFS (8.6 vs. 6.6 months, *p* = 0.017) and mOS (23.8 vs. 19.5 months, *p* = 0.042) in the maintenance group were better than in the intermittent group. Several studies (Wasan et al., 2014; Hegewisch-Becker et al., 2015; Koeberle et al., 2015; Simkens et al., 2015) showed that, after the standard first-line treatment for colorectal cancer patients, maintenance treatment could not prolong the OS, but could prolong the PFS in patients compared with discontinuation. Furthermore, a meta-analysis of six publications searched in PubMed, Embase, and CNKI with a population of 1975 mCRC patients concluded that compared with the maintenance, the first-line chemotherapy that was completely stopped until disease progression did not benefit mCRC patients in terms of OS and PFS (Ji et al., 2019). Therefore, for advanced colorectal cancer patients who benefit from the standard first-line treatment, the intermittent strategy is not recommended.

### 3.3 Continuous Treatment vs. Intermittent Treatment

In the MRC COIN trial (Adams et al., 2011), patients in group A were continued with induction regimen treatment until disease progression, and medication was stopped after induction treatment in group B. The results indicated that both PFS and OS in group B were shorter than in group A. The results suggest that discontinuation could only be selected for a few patients, such as patients with intolerable toxicity.

**FIGURE 3:**
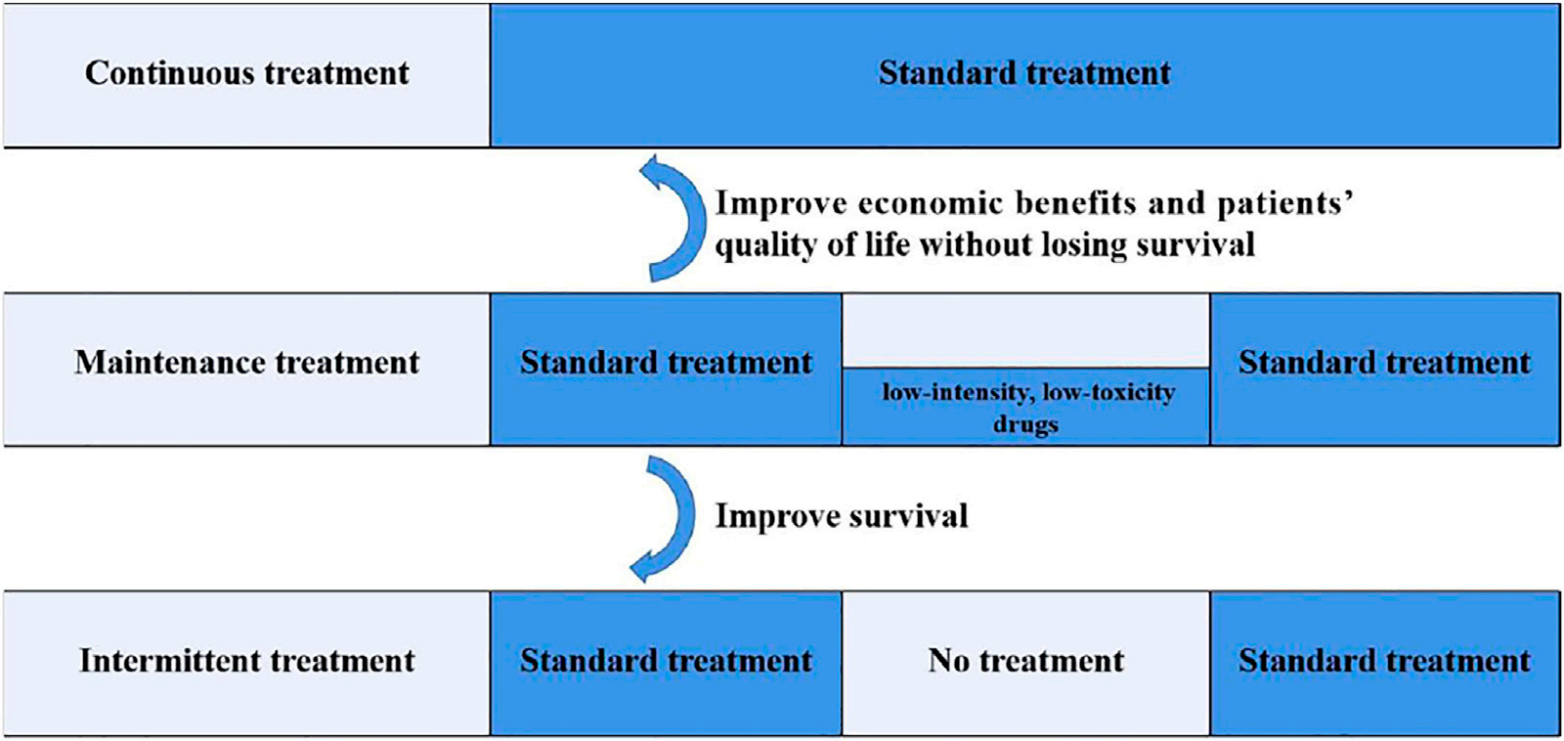
Three treatment patterns after the standard first-line treatment for mCRC. Maintenance therapy refers to the continuation of low-intensity and low-toxicity drugs after a period of first-line treatment, when the optimal efficacy is achieved and the disease is in a stable state. Continuous treatment means standard treatment until disease progression or intolerable toxicity. Intermittent treatment refers to complete discontinuation of all treatments after a period of standard treatment. Compared to continuous treatment, maintenance therapy can improve the economic benefits and patients’ quality of life without losing survival. Compared to intermittent treatment, maintenance therapy can improve survival.

Based on the abovementioned studies, maintenance therapy is necessary for mCRC patients who benefit from the standard first-line treatment, which can achieve the aims of prolonging the PFS, reducing adverse reactions, delaying the time of recurrence of tumor-related symptoms, and improving the patients’ quality of life. Domestic and foreign experts have reached a consensus on this (Xu et al., 2016; Yoshino et al., 2018; Benson et al., 2021). All the three treatment patterns are detailed in Figure 3. Recently a systematic review and network meta-analysis of 12 relevant randomized clinical trials comprising 5,540 mCRC patients considered that a maintenance strategy with fluoropyrimidine, with or without the combination of bevacizumab, is preferred. However, due to the lack of a certain OS benefit, shared decision making should include observation as an acceptable alternative (Sonbol et al., 2020).

### 4 Research Progress of Anti-EGFR mAb in mCRC Maintenance Therapy

Maintenance treatment is a commonly used treatment model in patients with advanced colorectal cancer who benefit from the standard first-line treatment, which can achieve the purpose of prolonging the PFS, reducing adverse reactions, delaying the time of recurrence of tumor-related symptoms, and improving the patients’ quality of life. However, it is still controversial whether mCRC can be applied with cetuximab for clinical maintenance therapy. Studies have not confirmed the advantages of maintenance therapy with anti-EGFR mAb, compared to discontinuation, bevacizumab maintenance therapy, and continuous therapy, so the role of anti-EGFR mAb in maintenance therapy remains uncertain. Furthermore, the long-term sustenance of anti-EGFR mAb therapy may induce secondary resistance, which is responsible for the limitation of anti-EGFR mAb in maintenance therapy.

## 4.1 Maintenance Therapy With a Single Anti-EGFR mAb

In 2012, the NORDIC VII trial (Tveit et al., 2012) compared the efficacy of cetuximab combined with the continuous or intermittent FLOX regimen (oxaliplatin 85 mg/m^2^ d1 at week 1, 3, and 5, leucovorin 500 mg/m^2^ every week for 6 weeks, and fluorouracil 500 mg/m^2^ every week for 6 weeks) in the first-line treatment of mCRC. Patients were randomized into three groups: the continuous FLOX + cetuximab group, continuous cetuximab combined with intermittent FLOX group (cetuximab maintenance group), and FLOX alone group. The final results showed that cetuximab combined with the FLOX regimen could not bring significant benefits, and the efficacy was not affected by the KRAS status, but there was no significant difference in mPFS and mOS among the three groups, suggesting that cetuximab maintenance treatment may play a certain role.

The Nordic-7.5 trial (Pfeiffer et al., 2015) is a supplement to Nordic VII, which aims to explore the feasibility of maintenance of cetuximab. A total of 152 cases of KRAS wild-type mCRC were included in the study. After eight cycles of the Nordic FLOX regimen combined with cetuximab (500 mg/m^2^, once every 2 weeks), patients of objective response continued to receive maintenance treatment of cetuximab once every 2 weeks, until the disease progresses or becomes intolerable. The results showed that the ORR was 62%, DCR was 88%, mPFS was 8.0 months, and mOS was 23.2 months in all of these 152 KRAS wild-type mCRC patients. This study suggests that, for KRAS wild-type mCRC patients, it is safe to receive maintenance treatment with cetuximab once in every 2 weeks, which can improve the survival time further. The MACRO-2 study (Aranda et al., 2018), a phase II study enrolling KRAS wild type only, compared the continuation of mFOLFOX6 (oxaliplatin 85 mg/m^2^ d1, leucovorin 400 mg/m^2^ d1, and fluorouracil 400 mg/m^2^ bolus d1 and then 2,800 mg/m^2^ civ 46 h) + cetuximab with maintenance cetuximab after eight cycles of mFOLFOX6 + cetuximab induction. A total of 193 patients were included in the study, including 129 in the cetuximab maintenance treatment group and 62 in the continuous mFOLFOX6 + cetuximab treatment group. The results showed that the main end point, PFS at 9 months, of the cetuximab maintenance treatment group was not inferior to that of the mFOLFOX6 + cetuximab continuous treatment group, which was 60 and 72%, respectively. The difference was not statistically significant, and peripheral neurotoxicity was significantly lower in the cetuximab maintenance treatment group (500 mg/m^2^ for every 14 days) than in the continuous treatment group. In COIN-B (Wasan et al., 2014), which is also a phase II study, 132 patients with KRAS wild-type mCRC treated with cetuximab plus mFOLFOX-based chemotherapy were randomized into two groups; one group was discontinued observation, and the other group was continued cetuximab maintenance treatment. The results showed that the primary endpoint, 10-month failure-free survival, was 50% in the observation group and 52% in the cetuximab maintenance treatment group, and PFS was 12.2 and 14.3 months in the two groups, respectively, and adverse reactions were all in the tolerable range. The study considered cetuximab maintenance (500 mg/m^2^ for every 14 days) treatment as the alternative scheme, but it needs to be confirmed by phase III studies. MACBETH (Cremolini et al., 2018), a phase II multicenter open randomized controlled study, compared 143 patients with RAS/BRAF wild-type mCRC who had received induction chemotherapy with cetuximab [intravenous (IV) dose of 500 mg/m^2^ over 60 min] combined with modified FOLFOXIRI (mFOLFOXIRI, consisting of irinotecan 130 mg/m^2^ d1, oxaliplatin 85 mg/m^2^ d1, L-leucovorin 200 mg/m^2^ d1, and fluorouracil 2,400 mg/m^2^ civ 48 h) every 14 days for up to 8 cycles, and then, they received maintenance treatment with cetuximab (500 mg/m^2^ IV over 60 min) and bevacizumab (5 mg/kg IV over 30 min), respectively. The primary endpoints, the 10-month PFS rates, of the two groups were 50.8 and 40.4%, respectively (*p* > 0.05). Although neither maintenance therapy approach met the primary endpoint, the investigators believed that a 4-month induction with mFOLFOXIRI combined with cetuximab was feasible and showed a high rate of surgical resection with this regimen. Hu et al. (2020) reported a retrospective exploration of targeted maintenance therapy in advanced colorectal cancer, which was based on the background of a Chinese patient assistance program. A total of 143 patients were assigned to the cetuximab (*n* = 79, 500 mg/m^2^ every 14 days) or bevacizumab (*n* = 64, 5 mg/kg every 14 days) groups. The mPFS was not significantly different between the Cet group and the Bev group: 5.9 months (95% CI 2.30–9.50) vs. 7.0 months (95% CI 3.69–10.31) (HR 1.17, 95% CI 0.77–1.79, *p* = 0.45).Thus, the authors suggest that maintenance therapy with cetuximab or bevacizumab can be considered an appropriate option following induction chemotherapy for the selected mCRC patients.

### 4.2 Maintenance Therapy of Anti-EGFR mAb Combined With Fluorouracil

From the abovementioned findings, we can see that anti-EGFR mAb maintenance therapy has a certain clinical significance, and fluoropyrimidine maintenance has also been confirmed in previous studies. So, can anti-EGFR mAb combined with fluoropyrimidine drugs can achieve better results? The Valentino study initiated by Pietrantonio et al. (2019) sought to answer this question, which was an open-label randomized phase II II study comparing panitumumab monotherapy with panitumumab + 5-FU/LV for maintenance therapy. A total of 229 patients with RAS wild-type previously untreated unresectable mCRC and adenocarcinoma were enrolled in the study. They were randomly divided into two groups and given eight cycles of panitumumab + FOLFOX4 (panitumumab, 6 mg/kg, oxaliplatin, 85 mg/m^2^ at day 1, leucovorin calcium, 200 mg/m^2^, and fluorouracil, 400 mg/m^2^ bolus, followed by 600 mg/m^2^ continuous 24 h infusion at days 1 and 2, every 2 weeks) induction therapy followed by sequential panitumumab (6 mg/kg) + 5-FU/LV (group A, 117 patients) or panitumumab (6 mg/kg) monotherapy (group B, 112 patients). The study used a noninferior design, and the results showed that the primary endpoint, the 10-month PFS rate in the intent to treat population, was significantly higher in group A than that in group B (combination schemes to be superior to panitumumab monotherapy maintenance), which was 59.9 and 49.0%, respectively, *p* = 0.01. It can be seen that the panitumumab + 5-FU/LV chemotherapy scheme is feasible for the maintenance treatment of patients with metastatic CRC. In addition, the SAPPHIRE study (Munemoto et al., 2019) from Japan randomized patients to the continuation group and panitumumab + 5-FU/LV maintenance treatment group after six cycles of the panitumumab + mFOLFOX6 treatment regimen. The results showed that the 9-month PFS rate was comparable in the continuation group and maintenance group (46.4 vs. 47.4%), while the incidence of adverse reactions in the maintenance treatment group was lower. The study considered the maintenance treatment with panitumumab + 5-FU/LV was feasible similarly. Capecitabine belongs to a fluorouracil class, and oral capecitabine combined with an anti-EGFR mAb as maintenance therapy may be an alternative after induction chemotherapy combined with anti-EGFR mAb in RAS/BRAF wild-type mCRC patients, which is more convenient compared to 5-FU. However, studies (Heinemann et al., 2014; Hagman et al., 2016; Qin et al., 2018) reported that the AEs, including diarrhea, rash, acne-like, and hand-and-foot syndrome, raised concerns about the safety of this protocol. Therefore, maintenance therapy with the combination of capecitabine and anti-EGFR mAb was highly controversial previously. To assess the biological activity and safety of capecitabine combined with cetuximab as a novel maintenance therapy for RAS wild-type mCRC, Professor Yuan et al. initiated a phase II prospective clinical trial (Wang et al., 2020). A total of 47 patients with RAS wild-type mCRC were enrolled. After 8–12 cycles of fluorouracil-based chemotherapy combined with cetuximab, patients received a reduced dose of capecitabine combined with cetuximab as maintenance therapy. The results showed that the primary study endpoint, the median maintenance PFS, was 7.2 months (95% CI, 5.8–8.6), while the mPFS was 12.7 months (95% CI, 11.8–15.4), and the mOS was 27.4 months (95% CI, 21.4–35.5). This study was the first one to explore the maintenance regimen of capecitabine combined with cetuximab, which achieved better efficacy and safety, while the optimal maintenance dose of capecitabine suitable for the Chinese population was 900 mg/m^2^. Therefore, the 2021 Chinese Society of Clinical Oncology colorectal cancer diagnosis and treatment guidelines also removed the description for “the use of capecitabine in combination with cetuximab is not recommended.” The phase III CLASSIC study of maintenance therapy with cetuximab-combined capecitabine, initiated by Xu et al. at the Zhongshan University Cancer Center, is currently ongoing. This is an open-label, randomized, multicenter, phase III study in patients with RAS/BRAF wild-type mCRC, to compare the efficacy and safety of cetuximab plus capecitabine versus single agent cetuximab as maintenance therapy, the primary endpoint of this trial is mPFS from randomization to PD, and secondary endpoints include OS, safety, PFS (first line from treatment initiation to PD), and quality of life.

In addition, in a multicenter retrospective study of maintenance therapy after the first-line anti-EGFR mAb combined with two drug schemes in 355 left-sided RAS/BRAF wild-type mCRC patients, the mPFS in the 5FU/LV + anti-EGFR mAb, anti-EGFR mAb monotherapy, 5FU/LV, and nonmaintenance cohorts was 16.0 months (95% CI = 14.3–17.7, 86 events), 13.0 months (95% CI = 11.4–14.5, 56 events), 14.0 months (95% CI 8.1–20.0, 8 events), and 10.1 months (95% CI = 9.0–11.2, 136 events) (*p* < 0.001), respectively (Parisi et al., 2021). This study provides a realistic clinical strategy for clinicians regarding maintenance therapy after receiving two-drug first-line induction therapy based on anti-EGFR, but the retrospective nature of the study, inherent selection bias, untargeted data collection, and small sample size of the cohort are some limitations that may have influenced the results of this study.

In contrast to the abovementioned anti-EGFR mAb maintenance study, the control group design of the PANAMA-AIO KRK0212 study (Modest et al., 2022) orally reported at the 2021 ASCO annual meeting is more suitable for the clinical problem which hopes to solve, that is, whether there is an advantage to adding anti-EGFR mAb to maintenance therapy over fluoropyrimidine-based monotherapy maintenance modalities. This study is a multicenter, randomized, open phase III clinical study, which explored the difference in efficacy between 5-FU combined with panitumumab and 5-FU monotherapy maintenance after six cycles of first-line panitumumab plus oxaliplatin plus 5-FU in RAS wild-type patients. A total of 248 patients were included in the study; 125 patients were included in the 5-FU combined with panitumumab group, and 123 patients were included in the 5-FU monotherapy group. At the time of data cutoff, 218 patients had efficacy data, and the study met its primary endpoint; that is, mPFS on maintenance therapy was improved in the 5-FU combined with panitumumab group versus the 5-FU alone group, 8.8 vs. 5.7 month, *p* = 0.014; OS was not statistically different from previous maintenance studies, although improved by 3 months, with 28.7 and 25.7 months in the combination and monotherapy group, respectively (HR = 0.84, 95% CI, 0.60–1.18, *p* = 0.32). The findings have been published in the Journal of Clinical Oncology. Table 1 shows the studies mentioned in this article about the maintenance therapy of anti-EGFR mAb in RAS wild-type mCRC patients.

**TABLE 1:** Summary of studies about the maintenance therapy of anti-EGFR mAb in Ras wild-type mCRC.

GCP	Primary endpoint	Patients	Induction therapy	Maintenance treatment group vs. control group	PFS	OS	Reference
NORDIC Ⅶ	PFS	109	Cetuximab + FLOX	Cetuximab vs. blank group	7.5 (6.7–8.3)	21.4 (14.2–28.5)	Mizukami et al. (2017)
NORDIC 7.5	OR, PFS	152	Cetuximab + FLOX	Cetuximab vs. blank group	8.0 (7.5–8.9)	23.2 (18.1–27.4)	Pugh et al. (2017)
COIN-B	FFS	132	Cetuximab + FOLFOX	Cetuximab vs. intermittent treatment	12.2 (8.8–15.6) vs. 14.3 (10.7–20.4)	22.2 (18.4–28.9) vs. 16.8 (14.5–22.6)	Sartore-Bianchi et al. (2016)
MACRO-2	PFS	193	Cetuximab + FOLFOX	Cetuximab vs. induction scheme	8.7 (7.4–9.5) vs. 9.8 (7.2–12.6)	23.5 (18.9–27.7) vs. 26.6 (17.8–35.8)	Sunakawa et al. (2016)
MACBETH	PFR	143	Cetuximab + mFOLFOXIRI	Cetuximab vs. bevacizumab	13.3 (11.2–17.3) vs. 10.8 (9.3–13.9)	37.5 (32.0–NE) vs. 37.0 (30.0–NE)	Emburgh et al. (2014)
Yuan XL Research	PFS	47	Fluorouracil-based chemotherapy	Cetuximab + capecitabine vs. blank group	12.7 (11.8–15.4)	27.4 (21.4–35.5)	Tournigand et al. (2006)
VALETINO	PFS	229	Panitumumab + FOLFOX4	Panitumumab+5-FU vs. panitumumab	12.0 (10.4–14.5) vs. 9.9 (8.4–11.0)	—	Ferris et al. (2018)
SAPPHIRE	PFR	164	FOLFOX + panitumumab	FOLFOX + panitumumab vs. panitumumab + 5-FU	9.1 (95% CI, 8.6–11.1) vs. 9.3 (95% CI, 6.0–13.0)	—	Lee et al. (2021)
PANAMA	PFS	248	Panitumumab + FOLFOX	Panitumumab + 5-FU vs. 5-FU	8.8 (7.6–10.2) vs. 5.7 (5.6–6.0)	28.7 (25.4–39.1) vs. 25.7 (22.2–28.2)	Hegewisch-Becker et al. (2015)
Alessandro et al. Retrospective Evaluation	PFS	355	Two-drug chemotherapy + anti EGFR	5FU/LV + anti EGFR, anti EGFR, 5FU/LV vs. induction scheme	16.0(14.3–17.7), 13.0(11.4–14.5), 14.0(98.1–20.0) vs. 10.1 (9.0–11.2)	39.6 (31.5–47.7), 36.1 (31.6–40.7), 39.5 (28.2–50.8), vs. 25.1 (22.6–7.6)	Chibaudel et al. (2009)

## 5 Conclusion

For RAS/BRAF wild-type mCRC, especially for the left half, anti-EGFR mAb combined with chemotherapy is the standard scheme of first-line treatment, but the status of anti-EGFR mAb in maintaining treatment after the first-line treatment is controversial, mainly due to the lack of large-scale phase III prospective clinical trials, and the drug resistance caused by the long-term use of anti-EGFR mAb cannot be ignored. This article retrospectively analyzes the relevant literature on the first-line and post first-line maintenance treatment of anti-EGFR mAb, and we can see that more and more studies support the maintenance treatment of anti-EGFR mAb, but some researchers have raised the problems of high cost and drug resistance. However, with the emergence of new evidence and more accurate screening of treatment-dominant groups, maintenance therapy with anti-EGFR mAb monotherapy or anti-EGFR mAb combined with fluorouracil-based schemes after the first-line chemotherapy combined with anti-EGFR mAb may strive for more treatment opportunities, optimize treatment strategies and prolong treatment continuity, then finally, leads to more survival benefit for suitable patients.

### 5.1 Outlook

Currently, guidelines on mCRC consider that the maintenance strategy with fluoropyrimidine, with or without the combination of bevacizumab, is preferred. Observation is also an acceptable alternative due to the lack of a certain OS benefit. However, whether anti-EGFR mAb can be used for maintenance treatment remains controversial. Through this review, we have reasons to believe that maintenance therapy with anti-EGFR mAb monotherapy or anti-EGFR mAb combined with fluorouracil-based schemes is also a good choice after the first-line chemotherapy combined with anti-EGFR mAb therapy in mCRC patients. As for the primary and acquired resistance of anti-EGFR mAb inevitably happening during the treatment, we consider that more clinical trials are needed to explore more effective biomarkers, while a deeper definition of anti-EGFR mAb resistance mechanisms will facilitate the development of new therapeutic strategies to overcome resistance. Furthermore, we believe that the introduction of new drugs will bring more and more survival benefits to patients.
